# Indirect effects of climate change altered the cannibalistic behaviour of shell-drilling gastropods in Antarctica during the Eocene

**DOI:** 10.1098/rsos.181446

**Published:** 2018-10-31

**Authors:** Gregory P. Dietl, Judith Nagel-Myers, Richard B. Aronson

**Affiliations:** 1 Paleontological Research Institution, Ithaca, NY 14850, USA; 2 Department of Earth and Atmospheric Sciences, Cornell University, Ithaca, NY 14853, USA; 3 Department of Geology, St. Lawrence University, Canton, NY 13617, USA; 4 Department of Ocean Engineering and Marine Sciences, Florida Institute of Technology, Melbourne, FL 32901, USA

**Keywords:** cannibalism, climate change, consumer–resource interactions, durophagy, drilling predation, indirect effects

## Abstract

The fossil record from Seymour Island, Antarctic Peninsula, provides a record of biotic response to the onset of global climatic cooling during the Eocene. Using drilling traces—small, round holes preserved on prey shells—we examined the effect of a cooling pulse 41 Ma on the cannibalistic behaviour of predatory naticid gastropods. We predicted that cannibalistic attacks would decline in response to the cooling climate, reflecting reduced activity levels, energy requirements and constraints on the chemically aided drilling process of the naticids. Surprisingly, however, cannibalism frequencies did not change. This counterintuitive result is best explained by a sharp reduction in durophagous (shell-crushing) predation in shallow-benthic communities in Antarctica that also occurred as the climate cooled. Reduced durophagous predation may have created a less-risky environment for foraging naticids, stimulating cannibalistic behaviour. The change in the top-down control exerted by shell-crushing predators on naticids may have counteracted the direct, negative effects of declining temperatures on the predatory performance of naticids. Our results suggest that the long-term consequences of climate change cannot be predicted solely from its direct effects on predation, because the temperature can have large indirect effects on consumer–resource interactions, especially where risk-effects dominate.

## 1. Introduction

Growing evidence suggests that climate change is altering how species interact, especially through the effects of temperature on organismal physiology [1–3]. Predicting exactly how ecological interactions will be altered is challenging because the physiological responses to temperature change (e.g. shifts in metabolic rate, growth, activity, etc.) often vary widely among species [3]. Models of the thermal responses of consumer–resource interactions suggest that if predator and prey possess traits that have asymmetric responses to temperature, such as metabolic rate and body velocity (e.g. how fast a predator moves when foraging for its prey), then changes in interaction dynamics are likely to arise [4]. Although variation in species' thermal responses complicates predictions for many interspecific interactions, predicting the responses of intraspecific interactions such as cannibalism may be less problematic, because the consumer and the ‘resource’ possess traits that respond more or less identically to temperature. In such cases, the dynamics are predicted to unfold in the same qualitative manner but at an accelerated or decelerated pace, depending on whether the temperature was increased or decreased, respectively [4].

**Figure 1.**
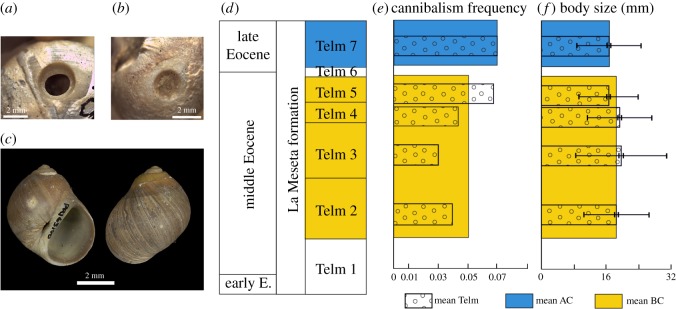
Close-up view of (*a*) complete naticid drillhole and (*b*) incomplete naticid drillhole. (*c*) Apertural (left) and abapertural (right) views of a specimen of *Falsilunatia* from the La Meseta Formation (LMF), Seymour Island, Antarctica. (*d*) Stratigraphic framework of the LMF. (*e*) Frequency of cannibalistic attacks before and after the Eocene cooling event. (*f*) Size distribution of *Falsilunatia* before and after the Eocene cooling event. BC, before climatic cooling; AC, after climatic cooling.

Cannibalism by shell-drilling naticid gastropods [5–9] offers an opportunity to test this prediction. Naticids are common predators in marine-benthic communities worldwide that leave characteristic round holes in the shells of their prey (figure 1*a,b*). Temperature should alter cannibalistic naticid interactions in predictable ways. For example, as temperature decreases, the activity levels (e.g. body velocities) of drilling predators decrease [10], which can reduce cannibalism by decreasing predator–prey encounter rates. It is also possible that drilling predators might not have to eat as often in the cold because of lowered metabolic requirements [11,12]. Changes in temperature are also thought to affect the naticids' shell-drilling process. Naticids drill holes through the shells of their prey mainly by chemical means to dissolve them, aided by mechanical rasping with the radula [12]. Because chemical reactions are slower at lower temperatures, it is likely that dissolution is retarded [13], which should increase the time required to complete an attack,^1^ reducing opportunities for future cannibalism [14].

Here, we test for the effect of declining temperature on the cannibalistic behaviour of the naticid *Falsilunatia* n. sp. (figure 1*c*) from the Eocene La Meseta Formation (LMF) on Seymour Island, Antarctic Peninsula. During the Eocene, the Southern Hemisphere was strongly influenced by climatic cooling. By the middle Eocene, approximately 41 Ma, temperatures had begun to fall, and at the Eocene/Oligocene boundary, approximately 34 Ma, the first continental-scale glaciers had formed in the Southern Hemisphere [15]. We predicted a decrease in cannibalistic attacks by naticids owing to the direct effects of temperature on predatory function as climatic conditions in Antarctica shifted from temperate to polar (e.g. [16]).

## 2. Stratigraphic and palaeoenvironmental setting

**Figure 2.**
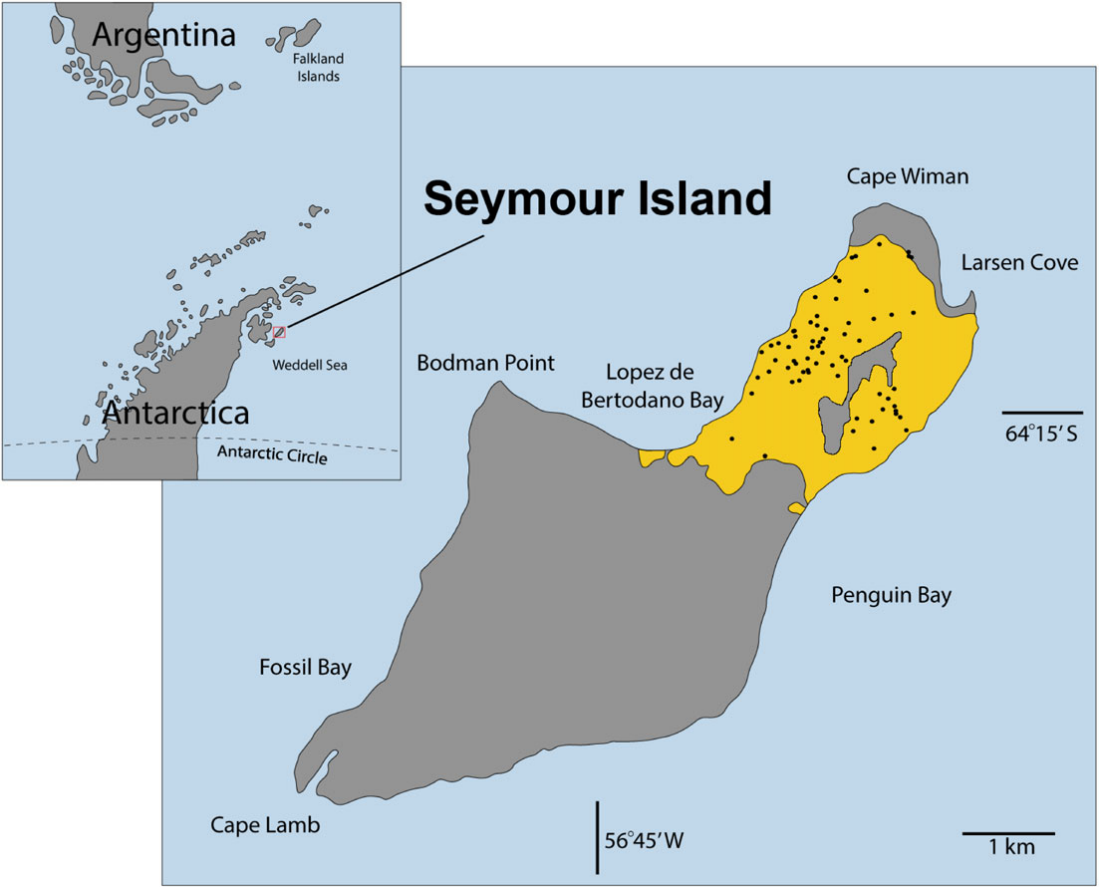
Map of Seymour Island with *Falsilunatia* sample localities (black dots) from the La Meseta Formation (yellow shading).

The LMF on Seymour Island, Antarctica (figure 2) is one of the most complete Eocene records in the world. The sedimentary succession consists of about 720 m of sandstones and mudstones interbedded with shell-rich, pebbly conglomerates [17]. The LMF is stratigraphically well-constrained and has been divided into seven lithofacies units: Telms 1–7 [18,19]. The sedimentary record is nearly complete except for one unconformity at the base of Telm 6 (e.g. [17]). The fossil assemblage of the LMF represents a shallow-water marine fauna inhabiting intertidal to subtidal environments. A significant facies shift occurs between Telms 5 and 6, indicating more freshwater influence in Telm 6 [20]. Stable oxygen isotope data from two benthic bivalves (*Cucullaea* and *Eurhomalea*) commonly found in the LMF are suggestive of about 10°C of cooling from the early Eocene climatic optimum (approx. 15°C; Telm 3) through the end of the Eocene (minimum approximately 5°C; Telm 7) [16].

## 3. Material and methods

### 3.1. Samples

More than 2000 well-preserved *Falsilunatia*^2^ specimens from 108 localities (figure 2) were examined for this study (see electronic supplementary material, S1, for raw data). Samples were grouped by stratigraphic units (Telms) and combined into two larger datasets, comprising the interval before climatic cooling (BC), represented by Telms 2–5, and after climatic cooling (AC), represented by Telm 7 (figure 1*d*). Considering the difference in environmental conditions, we excluded material from Telm 6 from the analysis. The examined material includes target-collected (*sensu* [25]) samples made by R. Aronson and D. Blake housed at the Florida Museum of Natural History, Gainesville, FL, USA and by W. Zinsmeister housed at the Paleontological Research Institution, Ithaca, New York, USA.

### 3.2. Data collection and analysis

To test our prediction that cannibalism should decline after the cooling event, complete and incomplete naticid drillholes in each sample of *Falsilunatia* were identified and counted. The frequency of cannibalism—defined as the proportion of prey that were attacked—was calculated as the number of specimens with drillholes (complete and incomplete) divided by the total number of specimens in the sample [9]. We tested for differences in the frequency of cannibalism between BC and AC samples using a chi-square goodness-of-fit test. Because larger naticids are more likely than smaller naticids to be cannibals [6,8], we compared the mean body size of all drilled and undrilled specimens in the AC versus BC samples. We used a *t*-test to assess whether the estimates of cannibalism could have been biased by an overall shift in the mean body size structure of the naticid population.

## 4. Results

The frequency of cannibalistic attacks varied from 0.03 in Telm 3 to 0.07 in Telm 7 (table 1; figure 1*e*). Contrary to our prediction, the frequency of cannibalistic attacks was 0.05 BC in Telms 1–5 compared with 0.07 AC in Telm 7 (figure 1*e*), although the difference was not statistically significant (*χ*^2^ = 0.188, df = 1, *p* = 0.171). Incomplete drillholes (figure 1*b*) accounted for 17.1% (*n* = 20) of the total number of drillholes, with failed attacks being more common AC, though not significant statistically (*χ*^2^ = 2.68, df = 1, *p* = 0.102; table 1). The mean body size of all drilled and undrilled naticids was 18.0 mm ± 0.20 s.e. and 17.1 mm ± 0.42 s.e. before and after the cooling event, respectively, suggesting no significant change (*t* = 1.71, df = 2,071, *p* = 0.09; figure 1*f*).

**Table 1.** Summary of drilling data on *Falsilunatia* from the La Meseta Formation of Seymour Island, Antarctica.

stratigraphic position	# undrilled specimens	# drilled specimens (complete)	# drilled specimens (incomplete)	cannibalism frequency	proportion failed attacks
Telm 7	326	20	5	0.071	0.20
Telm 5	838	53	8	0.068	0.13
Telm 4	151	7	0	0.044	0.0
Telm 3	322	5	5	0.030	0.50
Telm 2	339	12	2	0.04	0.14

## 5. Discussion

Despite the potentially limiting effects of low temperatures on the predatory function of *Falsilunatia*, our results show that cannibalistic drilling frequencies did not decrease with climatic cooling in the LMF. The lack of decline in cannibalism could be linked to the overall shift in the composition and structure of the Antarctic benthos that also occurred as the Eocene climate cooled. By the end of the Eocene, the predatory activities of decapod crustaceans, teleostean fishes and neoselachian sharks were substantially reduced [11,26,27]. Ultimately, asteroids, nemertean worms and other slow-moving predators replaced them as the top predators of the contemporary Antarctic benthos [27,28].

Populations of naticids might have benefited directly from the reduced predation pressure that accompanied climatic cooling. Ecological experiments have shown that increases in temperature may strengthen indirect species interactions in food webs. For instance, Miller *et al*. [29] found that the effects of predation risk and elevated temperature together suppressed foraging in the rocky intertidal by an intermediate-level, shell-drilling predator, *Nucella lapillus*, by more than half, suggesting that warming may enhance the top-down forcing effects of predation. Our drilling data for *Falsilunatia* are consistent with this idea, though in reverse: the disappearance of shell-crushing predators as the Eocene climate cooled may have relaxed shell-crushing predation pressure (i.e. top-down control) on naticids, making their environment less risky. The absence of predation pressure from shell-crushing predators may have subsequently stimulated naticid foraging, increasing cannibalism despite a reduced capacity for activity and metabolic scope at lower temperatures (as is the case today for other Antarctic marine invertebrates adapted to life in the cold [13]). This indirect effect of the reduced role of shell-crushing predators probably offset the expected decrease in cannibalism in colder environments represented by the upper section of the LMF.

The temporal pattern of incomplete drilling initially does not appear to support this interpretation. All else being equal, failed cannibalistic attacks should have been more common before the climate cooled, when the potential interruptions of the time-intensive drilling process [5] by shell-crushing predators were more common. The proportion of cannibalistic attacks that were unsuccessful, however, increased slightly, albeit not significantly, after the climate cooled (table 1). The tendency for defensive traits, such as escape body velocity, to be under stronger selection pressure to maintain nearly optimal performance across a range of temperatures than are traits related to consumption, such as attack body velocity, may help explain this counterintuitive result [3]. In general, for ectothermic animals, ‘escapes and failed attacks may be more common at low temperatures because escape body velocity typically remains close to peak levels and is thus higher than attack body velocity’ [2, p. 81]. This difference in activation energies implies that the defensive traits of naticids should have been less sensitive to cooling temperature changes than traits related to foraging.

An alternative hypothesis to explain the unexpected patterns of cannibalism is that *Falsilunatia* were more abundant than other prey resources after the cooling event. We cannot test this idea directly, but research on naticid cannibalism in other systems suggests that fluctuations in naticid abundance and cannibalistic drilling rarely exhibit a positive correlation [7]. Thus, it is unlikely that the stable frequencies of cannibalism we observed are a simple consequence of increased *Falsilunatia* abundance after the climate cooled.

Nor does a lack of alternative prey seem likely to explain the cannibalism patterns. If prey resources were scarce and/or spatially or temporally restricted, naticids would be forced to cannibalize each other. The post-cooling benthic community of Telm 7, however, includes abundant bivalves (e.g. *Saxolucina*, *Eurhomalea* and *Mya*) and gastropods (e.g. *Chlanidota*; formerly *Sudonassarius*; [23]) that were well within the size range vulnerable to naticids, suggesting that cannibalism was not a last resort for naticids foraging in a low-food environment.^3^ Indeed, if our risk hypothesis is correct, drilling predation on other prey taxa should also not decline in the LMF after the cooling event. Consistent with this prediction, Aronson *et al*. [27] showed that declining temperatures did not alter the frequency of drilling predation by naticids on *Eurhomalea,* the most abundant genus of infaunal bivalves in the LMF (0.06 BC and 0.06 AC).

Temporal shifts in the size structure of the naticid population also cannot explain the lack of decline in cannibalism. Chattopadhyay *et al*. [8] showed that the predator–prey size ratio can control the frequency of cannibalism in naticids, with cannibalism being more frequent when mean body size increases, because larger, older naticids are more likely to be cannibals. However, the mean sizes of *Falsilunatia* were similar before and after the cooling event (figure 1*f*).

Potential biases also must be ruled out before accepting our finding that cannibalism frequencies did not change with climatic cooling. Given that the predatory activities of shell-crushing predators were substantially reduced after the cooling event [11,26,27], chief among these potential biases is the removal of undrilled shells by durophagous predation [30,31]. If successful predation by shell-crushing predators often destroyed prey shells and shell crushers were more important components of the benthic fauna BC, the cannibalism frequencies we estimated for Telms 2–5 might be exaggerated, possibly suggesting that cannibalism intensified after the climate cooled. However, Smith *et al*. [31] have shown that when drilling frequencies are low (approx. 0.05, as is the case in the present study) the potential magnitude of such a bias—if it exists—is extremely low (only shifting the observed drilling frequency by a few percentage points) under low to moderate levels of shell loss from durophagous predation, boosting confidence in our interpretations.

Finally, and more broadly, our data highlight that models of the species-level responses to recent climate change may be misleading if they only consider the direct effects of temperature on physiology. The indirect effects of strong consumer–resource interactions potentially play a large role. For such interactions, the indirect effects of climate change will probably be significant drivers of the structure and function of marine-benthic communities, as they appear to have been for naticid cannibals in Antarctica's distant past.

## Supplementary Material

Electronic Supplementary Material S1

## Footnotes

1 Predatory gastropods living in cold environments may take up to 20 times longer—weeks instead of days—to drill through the shells of their prey than species adapted to warm water environments [13].

2 The taxonomy of the family Naticidae from the La Meseta Formation is not yet fully resolved (see [21, appendix S2, p. 24]). The two most-abundant species in the LMF were designated as *Polinices* cf. *subtenius* and *P. marambioensis* by Stillwell and Zinsmeister [22]. However, Crame *et al.* [21], in their recent, comprehensive overview of the molluscan fauna of the LMF, including much of the material treated in this study and additional collections housed at the British Antarctic Survey, concluded that these species should be assigned to the genus *Falsilunatia* (figure 1*c*; see [23, p. 216], which is commonly found in Antarctic waters today [24]. Crame *et al.* [21] further stated that *P. marambioensis* is highly unlikely to be a distinct species from *P.* cf. *subtenuis*. Two other naticid taxa, *Eunaticina infrequens* and *Sinuber powelli*, were described by Stillwell and Zinsmeister [22]; however, only a single individual of each of these rare species was identified in the material we examined for this study. Thus, we are confident that the predatory drillholes we found on the shells of *Falsilunatia* n. sp. were made by conspecific cannibals.

3 The possibility that a lack of alternative prey may influence the frequency of cannibalism was recently raised by Brezina *et al.* [9] for *Falsilunatia* n. sp. (identified by the authors as *P. marambionensis*) from the upper part of the *Cucullaea* I Allomember (=Telm 5 in this study) of the LMF. They found cannibalism attack frequencies that were approximately 2−3 times greater than those reported in this study and speculated that ‘the low relative abundance of other possible prey … could have affected … naticid behaviour’ [9, p. 213]. However, the Brezina *et al.* [9] samples—unlike those used in this study—came from a tidal channel deposit composed of ‘densely packed shell concentrations’, which were dominated by specimens of *Falsilunatia* n. sp., with low abundances of other taxa.

## Data accessibility

Data on the specimens used in the study are provided in electronic supplementary material, S1.
